# Optimising Scar Management Intervention in the Case of a Head-and-Neck Burn for a Patient with a Learning Disability

**DOI:** 10.3390/ebj5030019

**Published:** 2024-06-25

**Authors:** Katie Spooner, Matthew Pilley, Liz Rose, Stephen Frost, Reena Agarwal

**Affiliations:** 1 Department of Maxillofacial Surgery, University Hospitals of Leicester, Leicester LE1 5WW, UK; katie.spooner@uhl-tr.nhs.uk; 2 Maxillofacial Prosthesis Clinic, University Hospitals of Leicester, Leicester LE1 5WW, UK; matthew.pilley@uhl-tr.nhs.uk; 3 Private Practice, The Leicester Sports Medicine Clinic, Leicester LE2 1XD, UK; lizrosehandtherapy@gmail.com; 4 School of Plastic Surgery, East Midlands Deanery, University Hospitals of Leicester, Leicester LE1 5WW, UK; reena.agarwal@uhl-tr.nhs.uk

**Keywords:** burn rehabilitation, burn therapy, scar management, microstomia, head-and-neck burn

## Abstract

Scars following burns can often prove complex to manage, particularly when crossing joints or special areas such as the head and neck, due to contractures. This case report discusses the individualised care and rehabilitation provided to a burn patient with a learning disability. The patient suffered both full and partial thickness burns equating to a total body surface area (%TBSA) of 7% of the face, neck, and anterior chest via the self-ignition of clothing. Acute treatment was provided at a regional burn unit followed by further in-patient care and rehabilitation at our burn facility. A motion rehabilitation instrument was employed to manage potential orofacial contracture; however, due to the patient’s impaired social functioning, this device was found to be unsuitable. Subsequently, a bespoke mouth-opening device replicating an ice lolly was fabricated utilising computer-aided design (CAD), enhancing the patient’s understanding along with encouraging independence. Microstomia was a risk in this case; however, this was prevented via the discussed regime, and successful patient rehabilitation was achieved.

## 1. Introduction

Burns can be caused by a wide array of factors, such as thermal, electrical, chemical, or radiation-related factors, all of which compromise the integrity of the skin and soft tissues [1]. Mortality rates associated with burns have been declining in recent decades due to developments in intensive burn management. Despite this, they remain a significant global health problem. Burns account for approximately 180,000 deaths globally each year and are one of the leading causes of disability-adjusted life years (DALYs) [2,3].

The head and neck constitute one of the most common sites where a burn injury can occur. Due to the social significance of the face, these injuries can be devastating, as they can lead to substantial psychological and functional morbidity [4,5]. Both social and psychological factors are known to have a significant influence throughout the course of the management and rehabilitation of a burn injury and were key elements in early modern plastic surgery under Archibald McIndoe and what would later become known as the Guinea Pig Club [6,7].

Post-burn scarring of the head, face, and neck can have an impact on a number of facial and oral functions and potentially result in severe damage to oral continence, ventilation, vision, and the ability to communicate [8,9,10]. For burns affecting any area, the aims of management are to achieve an aesthetically satisfactory scar and a respectable functional outcome. For burns affecting the head, neck, and face, we must especially strive to achieve these aims in accordance with a high standard, as even small areas of scarring in this region can have a noticeable impact on function and cosmesis. In order to yield aesthetic scars and a good functional outcome, the management of burns of the head-and-neck region depends upon a combination of multiple factors, including appropriate wound care, nutrition, and subsequent scar care. This management must commence immediately from admission and continue long after the initial injury has been inflicted, and it necessitates a wide multidisciplinary team [11,12,13].

In particular, perioral burns can lead to microstomia, or a reduction in the oral aperture. This occurs as perioral burns show a high propensity to contract, and this is thought to be secondary to the circumferential nature of the orbicularis oris muscle. Despite the problems that post-burn microstomia can cause, non-operative and splinting techniques are the preferred first-line treatments [14]. There are a wide variety of devices that have been described for use in treating microstomia. In a 30-year review of the relevant literature up to 2002, Dougherty and Warden identified 37 different devices that could provide intra-oral or extra-oral splinting. These devices can be divided into static and dynamic, as well as with respect to how stretch is applied: vertically, horizontally, or circumorally [15]. However, there is no generally accepted format for the delivery of rehabilitation therapy for patients with burns to the head, face, and neck, either throughout their acute hospitalisation or during their reintegration into the community [16,17].

The process of fabricating a custom-made mouth-opening device utilising computer-aided design (CAD) software (Materialise 3Matics Design version 14.0) and three-dimensional (3D) model printing will be discussed. The process described allows for the optimisation of patient adherence by encouraging independence while simultaneously to accounting for a learning disability, with the aim of preventing microstomia.

### Literature Review

A PubMed search for ‘burns’ and ‘microstomia’ and ‘3D-printed’ and ‘computer-assisted design’ (or (CAD)) returned no results. A PubMed search for ‘burns’ and ‘microstomia’ and ‘prosthesis’ or ‘prosthetic’ returned 27 results.

There is no universally accepted protocol for the rehabilitation for burn-related microstomia; however, two surveys did review the practices of some therapists. Serghio et al.’s survey concerning North America showed that 43% of the respondent therapists preferred traditional static horizontal splints, with 24% preferring dynamic splints and 10% preferring circumferential splints. Another 10% reported a combination of using dynamic splint in the day and static splints at night [18]. Clayton et al. undertook a survey in Australia and New Zealand in 2010, repeated in 2014, and showed an increase in the proportion of therapists using a consistent regime in splinting and exercise, rising from 50% for both in 2010 to 61% and 74%, respectively, in 2014. Despite this, there was no obvious consensus in the content of the regime, other than the notions that the regime should ideally be administered 5 times per day and that custom splints were popular [19].

Oosterwijk et al. undertook a systematic review of burn scar contracture in 2017 and identified a prevalence of post-burn scar contractures of between 38 and 54% [20]. In a 2023 study of 1865 patients with burn injuries requiring surgical intervention and reconstruction, Goverman et al. identified that one-third (33%) of these patients developed at least one contracture at the point of hospital discharge [21].

There is no identifiable literature that defines a percentage risk or prevalence of microstomia following burns to the head, face, and neck, nor is there described a percentage or quantifiable risk after trauma or surgery with respect to this area of the body.

With regard to the rehabilitation of microstomia, a review of the literature was conducted through a PubMed search. From this, no articles were identified that discussed specifics of occupational/prosthetic management of microstomia in this patient cohort of learning disabilities with head-and-neck burns, either with or without the use of 3D printing or CAD. A single case report was identified regarding a child with facial injuries, both intra-oral and extra-oral, secondary to a firework explosion. In this case, a static intra-oral device was developed and used in addition to mouth-opening exercises [22]. With regard to CAD, its use has been documented for a variety of medical uses, including maxillofacial implants and external prostheses, extracellular matrices for soft-tissue defects, and, perhaps of the most relevance, hypertrophic scar management with custom-fitted silicone sheets [23,24,25,26].

A 3D-printed orthosis that was adjustable and allowed static horizontal or vertical stretching has been described by Almendinger [27]. However, there is currently no other report that we have been able to identify in the literature that describes a process utilising CAD and 3D printing in the fashion that we will present here.

Microstomia is a qualitative diagnosis that consists of a reduction in the oral aperture, but there is no quantitative description of microstomia, which may present a barrier to the standardisation of reporting. However, most papers discuss measures of vertical and horizontal mouth opening, and a patient-reported functional assessment has been described by Couture et al. This assessment, the mouth impairment and disability assessment (MIDA), seeks patient and therapist reports for various aspects of their function, including disability index, patient satisfaction, social impact, and associated symptoms [28].

## 2. Patient Information

A 53-year-old female presented to a regional burn unit following full- and partial-thickness flame burns to the anterior and lateral aspect of the face, neck, and anterior chest after a self-immolation injury. The patient was known to suffer from a number of learning disabilities and had a history of depression. She required full-time support from her elderly parents prior to the injury. The patient was independently mobile but had a sedentary lifestyle, enjoying music, teddy bears, and attending religious events.

## 3. Clinical Findings

The patient’s burn injury was determined to constitute 7% of her total body surface area (%TBSA). Acute treatment in the Burn Unit included intubation and the debridement of the burnt areas using NexoBrid^®^ proteolytic gel. After extubation and stabilisation, the patient was sent to the local burn facility for further in-patient treatment and rehabilitation. Given the distribution of injuries, there was concern that a dysfunction of the oral aperture, such as microstomia, could develop secondary to the contracture of the patient’s scars. As such, a referral was made to the Maxillofacial Prosthetics Department for the provision of a mouth-opening device in an attempt to prevent microstomia.

**Figure 1:**
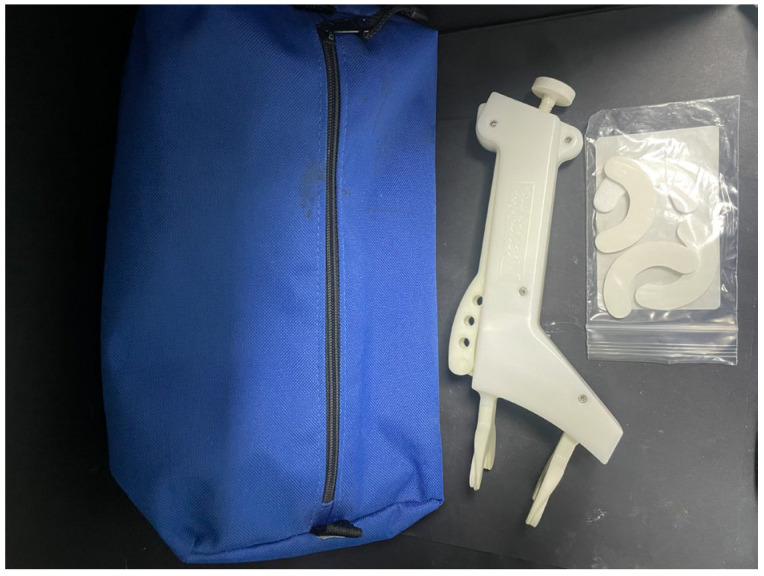
The TheraBite^®^ device employed.

A TheraBite^®^ device was initially provided to the patient in order to address the concerns of microstomia (Figure 1). A TheraBite^®^ device has a built-in cushioned mandible mouthpiece that opens once pressure is applied to the hand aid, activating the stretching functionality [29]. Despite studies stating that the TheraBite^®^ device is easy to use [30,31], the patient in this case found it very difficult to operate independently, so a more bespoke solution was required. To achieve this, a decision was made to design a custom-made mouth-opening device that would resemble something more familiar to the patient in order to encourage independence and adherence. After a discussion with the patient and her family, it was decided that a device resembling an ice lolly would be most suitable for this particular case.

## 4. Therapeutic Intervention—Design and Implementation

In order to develop the custom device, an ice-lolly maker was attained in order to acquire the shape of the proposed mouth-opening device and duplicated using an irreversible hydrocolloid impression material. This impression material went on to be cast using a type 3 dental stone.

**Figure 2:**
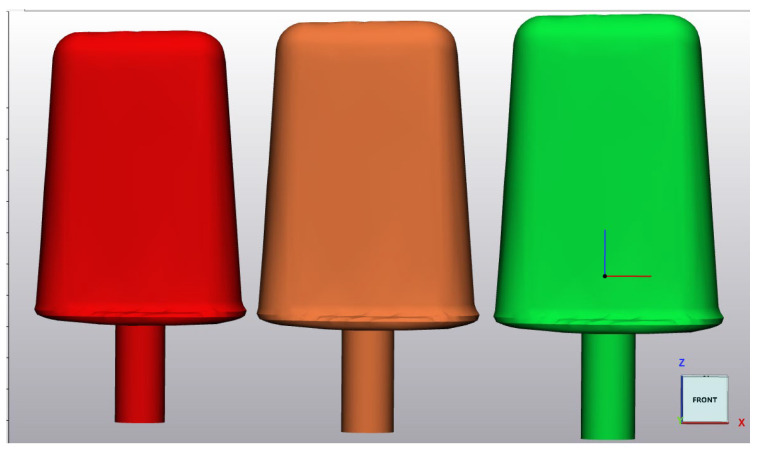
Illustrates the three ice-lolly sizes in CAD software.

The cast was then cone-beam-scanned, downloaded, and converted into a stereolithography file (STL) in Materialise Mimics—a 3D medical image segmentation software product. The STL file was then transferred to the Materialise 3-matic design optimisation programme, which allowed for the size of the “ice-lolly” to be manipulated consecutively. This allowed for the creation of three differently sized “ice-lollies” deemed to be best suited to the case (52 mm, 58 mm, and 63.5 mm wide) (Figure 2). Of note, for females, the inter-commissural distance has been described as ranging between 5.53 cm and 7.77 cm [32] and is generally related to height.

These figures guided the planned “ice-lolly” sizes during the design process. The “ice-lollies” were then 3D-printed and invested into dental stone using a large investing flask to form the moulds for the final devices. Once the stone was set, the 3D printed “ice-lollies” were removed from the investing flasks. A separating medium was then applied to each mould and then packed and cured using both pink and yellow heat-cured dental acrylic. This was performed to ensure the devices would resemble different ice-lolly flavours. After curing, the mould was opened, and the “ice-lolly”/mouth-opening devices were trimmed, rubbered, polished, and, finally, decontaminated before they were employed for patient care. The STL files used are provided in Supplementary Files for this paper.

Whilst the design of these devices corresponds to a serial static orthosis [33], the increase in width along the length of the device, along with the intention of appearing like and being utilized like an ice-lolly, with inward and outward movement, the end result allows for a dynamic stretch effect to be achieved with each device, if desired.

### 4.1. Small Sized Custom-Made Mouth-Opening Device

**Figure 3:**
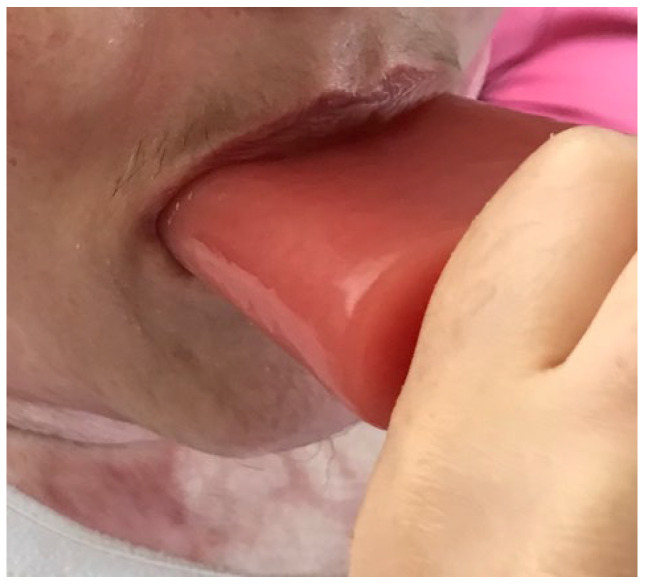
Illustrates the use of the small custom-made mouth-opening device.

The smallest custom-made mouth-opening device was issued to the patient in July 2020, whilst she was still an in-patient (Figure 3). Emotional support and humour were used to encourage patient adherence, alongside maintaining consistency between health care professionals. This was carried out to avoid confusion that may have instigated distress for the patient. During this time, mouth measurements were taken: 4.7 cm (vertical) and 7.5 cm (horizontal). The timings initially achieved were three sets of 3 min.

### 4.2. Medium-Sized Custom-Made Mouth-Opening Device

**Figure 4:**
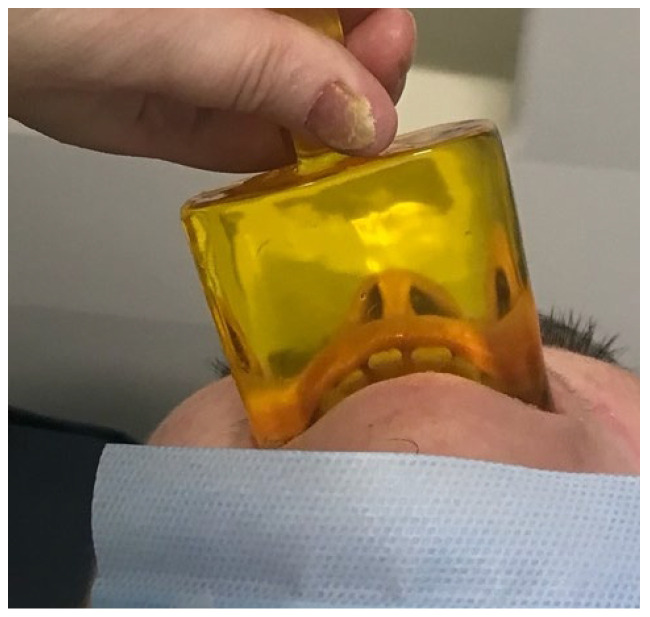
The use of the medium-sized custom-made mouth-opening device whilst encouraging the natural full extension of the neck.

The medium-sized custom-made mouth-opening device was issued to the patient six weeks later, in September. As has been discussed in the literature, the patient was also intermittently positioned on her back without a pillow in order to encourage the natural full extension of her neck (Figure 4) [34].

During the course of using the medium-sized mouth-opening device, the patient was discharged from in-patient care and subsequently contacted by both specialist nursing and occupational therapy teams consecutively in the community.

### 4.3. Large-Sized Custom-Made Mouth-Opening Device

**Figure 5:**
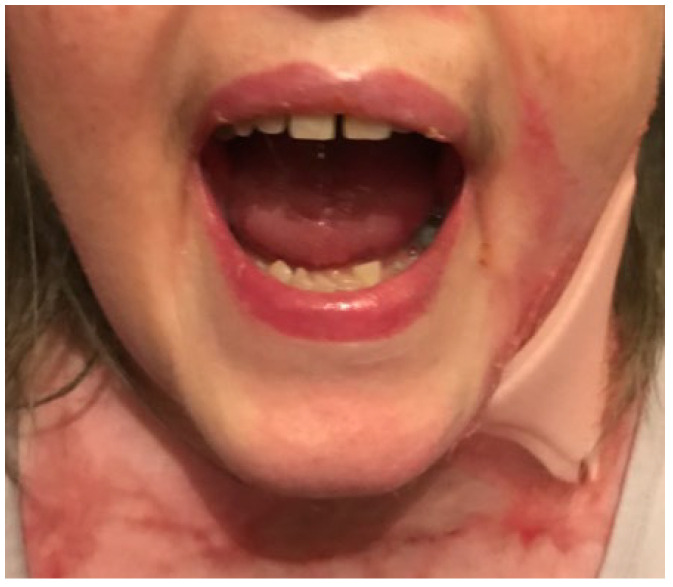
Illustrates the successfully rehabilitated patient.

The large-sized custom-made mouth-opening device was provided to the patient fourteen weeks later, in December. This device was used successfully at home until her discharge three months later. All mouth-opening devices utilised in this case were used for a period of 5 min three times per day to achieve the successful rehabilitation of this patient, as illustrated in Figure 5. This regime was prescribed as it was felt to be achievable for this patient, but it was not based on any pre-defined protocols. The patient adhered to her stretching treatment well throughout her care. Unfortunately, due to the patient’s learning difficulties and the complexity of the previously reported MIDA, we were unable to collect any reliable quantitative feedback from this patient.

## 5. Follow-Up and Outcomes

After discharge, the patient had returned to her usual, pre-injury ability to undertake feeding, oral hygiene, and speech, in addition to eating her favourite meals and singing without issue.

## 6. Discussion

Head-and-neck burns can be severely damaging injuries, and the aim in their acute and long-term management is to reduce complications that most often affect a patient’s psychological and functional health. Skin contracture is one of the main long-term complications associated with burns, in addition to other forms of pathological scarring, such as hypertrophic and keloid scarring.

It is crucial to remember that burn contracture results from a combination of wound contracture, which occurs during the initial healing process, and scar contracture, defined as shrinkage of an already-healed scar. The former occurs primarily in response to the action of fibroblasts, while the latter appears to be principally affected by myofibroblasts. It has also been suggested that some aspects of pathological scarring may be related to the abnormal modulation or, rather, lack of downregulation of myofibroblasts [35,36]. This theory could explain the variation in contracture that has been observed between patients. In the case presented here, our intervention was acting primarily on the latter phase of burn contracture; i.e., our patient’s wounds had healed by the time the custom, 3D-printed mouth-opening device was employed.

Deep burns to the face can lead to the contracture of the perioral tissues, causing a significant loss of the ability to open one’s mouth, commonly known as microstomia. Microstomia can be painful, and it can make speech difficult and limit the movement of the mandible. In more severe cases, oral and dental hygiene can become compromised, and feeding may be jeopardised. As discussed above, it is theorised that it is the relation of the injury to the orbicularis oris muscle that predisposes contracture in perioral burns [14]. It is also accepted that the application of mechanical force to and the stretching of scars can contribute to the development of pathological scarring [37]. This may explain why the perioral skin, which undergoes a high degree of movement during eating and speech, may be prone to contracture and hence an area to monitor for post-burn contractures.

Goverman et al.’s paper on post-burn contractures also identified the frequency of microstomia, placing at 0.27%, although this is a percentage of all the patients in the study and does not quantify the number of patients with burns to the head and neck that would naturally be at risk of microstomia [21].

Historically, a range of modalities have been utilised for the prevention of microstomia, including tongue blades, encouraging a patient to eat uncut fruit, and/or the use of an objective measuring technique, thus encouraging patient involvement [38,39,40]. Similarly, a range of intraoral and extraoral devices that produce stretching motions, focusing on vertical, horizontal, and/or circumoral directions, have also been used and can allow static or dynamic stretching [15,41,42,43]. The exact mechanism by which stretching improves pathological scarring is not well understood, but there is a theorised explanation stating that the stretching forces can disrupt fibrotic tissue, increase laminin and collagen content, and so result in improved pliability of the tissues [44].

All known devices and techniques of stretching were determined to be unsuitable for this patient in light of her learning disability. Therefore, through the use of the innovation of 3D printing and CAD and the fabrication of bespoke, patient specific mouth-opening devices, patient engagement was achieved, and microstomia was successfully prevented.

## 7. Conclusions

This case report demonstrates the successful implementation and utilization of a bespoke mouth-opening device, fabricated utilising CAD, in a burn patient’s treatment. The use of this technique has assisted in preventing the formation of problematic scar tissue and the contracture of the perioral tissues, thus avoiding microstomia. The use of this bespoke mouth-opening device supported the patient’s dexterity and prevented confusion whilst simultaneously encouraging independence, eliminating distress and optimising adherence to the rehabilitation regimen. This engagement was crucial for a successful rehabilitation.

There are a number of limitations to this report. The first is that no formal evaluations were used before or after intervention. As a result, validation of this intervention is necessary through the standardized assessment of impact across a larger cohort. This will allow accurate determination efficacy and facilitate generalisability. The authors recognise that the distribution of these injuries was not circum-oral, but this patient is presented in order to provide a proof-of-concept demonstration of this mouth-opening device. In addition, this patient was sent to the authors’ hospital, with acute management delivered by a team in another unit, for which we did not have an input.

To the best of our knowledge, this is the first reported use of CAD to develop a suitable device to support in the management of microstomia or perioral burns in a patient with a learning disability. The authors hope that this case and the described process can inspire colleagues dealing with patients with similar problems to utilise a combination of CAD, 3D printing, and personalised design in the devices used in the treatment of their patients.

## Data Availability

Data are contained within the article and Supplementary Materials.

## Supplementary Materials

The following supporting information can be downloaded at: https://www.mdpi.com/article/10.3390/ebj5030019/s1, S1: STL files for reproduction of the described device.
